# Synovial and Systemic Pharmacokinetics of Florfenicol and PK/PD Integration against *Streptococcus suis* in Pigs

**DOI:** 10.3390/pharmaceutics14010109

**Published:** 2022-01-03

**Authors:** Zoltán Somogyi, Patrik Mag, Dóra Kovács, Ádám Kerek, Pál Szabó, László Makrai, Ákos Jerzsele

**Affiliations:** 1 Department of Pharmacology and Toxicology, University of Veterinary Medicine, István Utca 2, H-1078 Budapest, Hungary; Somogyi.Zoltan@univet.hu (Z.S.); mag.patesz@gmail.com (P.M.); Kovacs.Dora@univet.hu (D.K.); Kerek.Adam@univet.hu (Á.K.); 2 MS Metabolomics Laboratory, Center for Structural Study, Research Center for Natural Sciences, Magyar Tudósok krt. 2, H-1117 Budapest, Hungary; Szabo.Pal@ttk.hu; 3 Department of Microbiology and Infectious Diseases, University of Veterinary Medicine, Hungária krt. 23-25, H-1143 Budapest, Hungary; Makrai.Laszlo@univet.hu

**Keywords:** florfenicol, pharmacokinetic, MIC, AUC, AUC_24h_/MIC, synovial fluid, swine

## Abstract

Florfenicol is a member of the phenicol group, a broad-spectrum antibacterial agent. It has been used for a long time in veterinary medicine, but there are some factors regarding its pharmacokinetic characteristics that have yet to be elucidated. The aim of our study was to describe the pharmacokinetic profile of florfenicol in synovial fluid and plasma of swine after intramuscular (i.m.) administration. In addition, the dosage regimen of treatment of arthritis caused by *S. suis* was computed for florfenicol using pharmacokinetic/pharmacodynamic (PK/PD) indices. As the first part of our investigation, the pharmacokinetic (PK) parameters of florfenicol were determined in the plasma and synovial fluid of six pigs. Following drug administration (15 mg/kg_bw_, intramuscularly), blood was drawn at the following times: 10, 20, 30, 40, 50 and 60 min, 2, 3, 4, 5, 6, 7, 8, 12, 24, 48 and 72 h; synovial fluid samples were taken after 1, 2, 3, 4, 6, 8, 12, 24, 48 and 72 h. The concentration of florfenicol was determined by a validated liquid chromatography-mass spectrometry (LC-MS/MS) method via multiple reaction monitoring (MRM) modes. As the second part of our research, minimum inhibitory concentration (MIC) values of florfenicol were determined in 45 *S. suis* strains isolated from clinical samples collected in Hungary. Furthermore, a strain of *S. suis* serotype 2 (SS3) was selected, and killing-time curves of different florfenicol concentrations (0.5 µg/mL, 1 µg/mL and 2 µg/mL) were determined against this strain. Peak concentration of the florfenicol was 3.58 ± 1.51 µg/mL in plasma after 1.64 ± 1.74 h, while it was 2.73 ± 1.2 µg/mL in synovial fluid 3.4 ± 1.67 h after administration. The half-life in plasma was found to be 17.24 ± 9.35 h, while in synovial fluid it was 21.01 ± 13.19 h. The area under the curve (AUC_24h_) value was 54.66 ± 23.34 μg/mL·h for 24 h in plasma and 31.24 ± 6.82 μg/mL·h for 24 h in synovial fluid. The drug clearance scaled by bioavailability (Cl/F) in plasma and synovial fluid was 0.19 ± 0.08 L/h/kg and 0.29 ± 0.08 L/h/kg, respectively. The mean residence time (MRT) in plasma and synovial fluid was 24.0 ± 13.59 h and 27.39 ± 17.16 h, respectively. The steady-state volume of distribution (V_ss_) in plasma was calculated from Cl/F of 0.19 ± 0.08 L/h/kg, multiplied by MRT of 24.0 ± 13.59 h. For the PK/PD integration, average plasma and synovial fluid concentration of florfenicol was used in a steady-state condition. The obtained MIC_50_ value of the strains was 2.0 µg/mL, and MIC_90_ proved to be 16.0 µg/mL. PK/PD integration was performed considering AUC_24h_/MIC breakpoints that have already been described. This study is the first presentation of the pharmacokinetic behavior of florfenicol in swine synovia as well as a recommendation of extrapolated critical MICs of *S. suis* for therapeutic success in the treatment of *S. suis* arthritis in swine, but it should be noted that this requires a different dosage regimen to that used in authorized florfenicol formulations.

## 1. Introduction

Antimicrobial resistance (AMR) is among the top health issues threatening humankind. AMR can be tackled most effectively via the *One Health* approach, which requires the joint action of human health professionals, veterinarians and the scientific society. Prudent antibiotic use in the animal industry contributes to reducing AMR and requires evidence-based (ideally a PK/PD approach) use of antimicrobials in each animal species.

Florfenicol is a broad-spectrum antibacterial agent [1] that has bacteriostatic activity through inhibiting protein synthesis of bacteria at the 50S ribosome subunit [2]. In porcine health management, florfenicol is primarily administered to treat bacterial respiratory infections [2,3,4,5,6]. It can also be used in bovine health for the treatment of septic arthritis, as supported by PK data [4,7]; however, no studies have been performed on pigs with this indication.

The bioavailability of florfenicol is good to excellent in most farm animals, but there may be differences between species and routes of administration. In pigs, bioavailability of the drug after i.m. administration can reach up to 96% [3,5,8]. The distribution of florfenicol is excellent, reaching high tissue concentrations in the lower respiratory tract [9]. The plasma protein binding of florfenicol is less than 20%, contributing to good tissue penetration [3,10]. There are no publications available regarding florfenicol PK in synovial fluid of pigs.

*S. suis* is a highly important infectious agent worldwide [3,11,12,13,14,15]; it can cause serious economic losses to the swine industry [3,14,16]. The most important clinical signs and lesions of the infection are septicaemia, meningitis and arthritis [3,11,12,13,14,16]. In the absence of antibacterial treatment, morbidity and mortality can reach high levels. *S. suis* arthritis causes economic loss and damage to animal welfare. Minimum inhibitory concentrations of florfenicol against *S. suis* strains are usually lower ≤2 µg/mL [17,18], meaning that, generally, this bacterium is sensitive to florfenicol, taking into account the relevant Clinical and Laboratory Standards Institute (CLSI) guidelines [19].

The purpose of PK/PD analysis is to establish a model that describes the efficacy of antibiotics against a certain pathogen in a certain target tissue or plasma at a specific dose [20,21,22]. The PK/PD integration approach for predicting the usefulness of florfenicol in the treatment of pig diseases caused by *S. suis* was described by Lei et al. [3]. The present study adopted similar methods to investigate the efficacy of florfenicol against arthritis of swine caused by *S. suis*.

The aim of the present study was to determine the plasma and synovial fluid concentration-time profile of florfenicol administered to swine intramuscularly at the authorized dose of 15 mg/kg_bw._ It has been published that florfenicol is present in the synovial fluid of cattle at therapeutic concentrations [7], which leads to the hypothesis regarding its application in swine joint infections. Furthermore, according to CLSI guidelines, florfenicol has breakpoints in respiratory tract infections of swine caused by *S. suis* if the MIC of florfenicol is equal to or lower than 2 µg/mL [19]. We therefore aimed to determine whether 15 mg/kg_bw_ of i.m. administered florfenicol could be effective in arthritis of swine caused by *S. suis*.

## 2. Materials and Methods

### 2.1. Experimental Animals

Six male pigs (Danish landrace x Yorkshire), 6 weeks of age, 12.8 ± 1.66 kg body weight (BW), were purchased from a local commercial pig farm. Animals were fully vaccinated and free from any clinical signs of bacterial or non-infectious disease. The pigs were kept at 21 °C, 70% relative humidity and a 12 h/day light cycle. There was one week of acclimatization before experimentation to ensure the absence of any residual drugs, such as antimicrobials and antiparasitics. Standard commercial feed, free from antibacterial and antiparasitic agents, and water were supplied ad libitum. The study was authorized by the Local Animal Welfare Committee of the University of Veterinary Medicine, Budapest, and by the Government Office of Pest County, Food Chain Safety, Plant Protection and Soil Conservation Directorate, Budapest, Hungary (decision number: PE/EA/288-7/2020).

### 2.2. Experimental Design

Florfenicol (Floron 300 mg/mL solution for injection 100 mL, Krka, d.d., Novo mesto, Slovenia) was administered intramuscularly at a dose rate of 15 mg/kg_bw_. Each dose was administered via intramuscular injection to the left of the neck. A 20G × 1 inch needle and appropriately sized syringes were used for administration. A blank sample of blood and synovial fluid was taken before drug administration. After drug administration, blood samples were taken at the following times: 10, 20, 30, 40, 50 and 60 min, 2, 3, 4, 5, 6, 7, 8, 12, 24, 48 and 72 h; synovial fluid samples were taken after 1, 2, 3, 4, 6, 8, 12, 24, 48 and 72 h. Analgesia and sedation was provided with tiletamine-zolazepam combination (3.5 mg/kg_bw_) (Zoletil, Virbac, Carros, France), xylazine (1.32 mg/kg_bw_) (Sedaxylan, Aurovet Animal Health B.V., Bladel, Netherlands) and tramadol (1.8 mg/kg_bw_) (Contramal, STADA Arzneimittel AG, Bad Vilbel, Germany) [23] before all arthrocentesis, as the intervention proved to be stressful and slightly painful for the animals. Yang et al. [24] and Rottbøll et al. [25] reported the disadvantages of anesthesia in PK determination on florfenicol; however, Wang et al. [26] demonstrated that anesthesia has a negligible effect on the PK properties of florfenicol. Additionally, in our study, the pigs were anesthetized only during synovial fluid sampling. Blood samples were taken by venipuncture from the jugular vein into heparinized vacutainers. Synovial fluid samples were taken by arthrocentesis from the carpal, tarsal and stifle joints alternately. The collected blood samples were centrifuged at 1482 *g* for 10 min to obtain the blood plasma. Blood plasma and synovial fluid samples were stored at −80 °C until subjected to chromatographic-mass spectrometric analysis.

### 2.3. Tandem Mass Spectrometry Analysis

A Sciex 6500QTrap tandem mass spectrometer (Sciex, Framingham, MA, USA) coupled with an Agilent 1100 HPLC system (Agilent Technologies, Waldbronn, Germany) was used for the quantitation of florfenicol. A Kinetex XB C18 (50 × 2.1 mm, 2.6 µm) column (Phenomenex) was applied for the separation of the target compound from the matrix components. Water containing 0.1% formic acid (eluent A) and acetonitrile containing 0.1% formic acid (eluent B) were used in gradient mode as follows: the initial composition contained 10% of eluent B, and this was kept for 0.5 min; then, B was increased to 90% at 2.5 min. This composition was held for 0.5 min and set back to the initial composition at 0.3 min. The equilibrating period was 2.2 min. The overall run time was 6 min. The flow rate of the mobile phase was 0.5 mL/min. The eluent from the LC was introduced into the mass spectrometer, where electrospray ionization was applied in positive ion detection mode. The spray voltage was 5000 V, the source temperature was 450 °C. The values of the curtain gas, evaporation gas and drying gases were 40, 40 and 35 arbitrary units, respectively. The multiple reaction monitoring (MRM) experiment was applied for the quantitation. The Q1/Q3 transitions were 358.2/241 and 358.2/170 in the case of quantifier and qualifier transitions, respectively. The collision energy was 35 eV. A 5-point calibration curve was used for the range of interest. The accuracy of the QC samples was 96–102%. The LOQ and LOD values of the method were 0.1 and 0.05 ng/mL, respectively. These values are far from the real concentration observed in our samples. The calibration samples were freshly prepared every day. Analyst 1.6.3 software was used for controlling and data processing.

### 2.4. Pharmacokinetic Analysis

The parameter values of PK were calculated from the plasma and synovial fluid florfenicol concentrations using PKSolver 2.0 (Nanjing, China). To select the appropriate PK models, the drug concentrations were recorded in semi-logarithmic graphs. Data from the 6 animals were individually analyzed by the non-compartmental method, and the mean value was calculated for each PK parameter. A recent paper demonstrated negligible protein binding at low concentrations, to a maximum of 5% at high concentrations, in cattle [27]. This confirmed low protein binding of the phenicol (less than 15%), determined in vitro in earlier investigations [28,29]. A similar result was obtained by Lei et al. [3]. Consequently, protein binding was ignored in our calculations. The variables calculated were maximum plasma concentration (C_max_), area under the curve for 24 h (AUC_24h_), area under the concentration-time curve from zero time to infinity (AUC_0-∞_), time of maximum concentration (T_max_), terminal half-fife (T_1/2_), drug clearance scaled by bioavailability (Cl/F), mean residence time (MRT) and steady-state volume of distribution (V_ss_). V_ss_ was calculated from the drug clearance scaled by bioavailability, multiplied by the mean residence time [30]. This was necessary to determine breakpoints of florfenicol against *S. suis* under steady-state conditions [31]. After a single dose of florfenicol (i.m., 15 mg/kg_bw_), the AUC_24h_ was equal to AUC_0-∞_ in steady-state condition. However, florfenicol did not reach equilibrium with this formulation after the first administration. Therefore, a loading dose was necessary, as calculated in Table 1. The target concentration equaled the average plasma concentration (C_24av_) and should be achieved with the loading dose and a concomitant maintenance dose (i.m., 15 mg/kg_bw_). Furthermore, the synovial fluid-to-plasma ratio was computed by AUC_0-∞_ of plasma divided by AUC_0-∞_ of synovial fluid in a steady-state condition and was used for PK/PD integration. These calculations are shown Table 1.

### 2.5. Determination of Minimum Inhibitory Concentration

The *S. suis* strains (n = 45) tested in this study were clinical isolates of Hungarian origin between 2018 and 2021. MICs of florfenicol against *S. suis* isolates were determined by broth microdilution. MIC_50_ and MIC_90_ values were computed as the MIC that inhibited the growth of 50% and 90%, respectively, of the isolates in different clusters. Determination of MIC and calculation of MIC_50_ and MIC_90_ were performed according to CLSI [19]. For each *S. suis* strain obtained, bacteria were cultured in Brain-Heart Infusion (BHI) liquid broth (Biolab co. ltd., Budapest, Hungary) at an ambient temperature of 37 °C for 24 h before the experiment. After growth, suspensions were centrifuged at 3000× *g* for 10 min and then washed with sterile physiological saline, centrifuged at 3000× *g* for 10 min again and resuspended in physiological saline. The optical density of the suspensions at 600 nm was set to 0.1 (OD_600_ = 0.1), with the appropriate amount of physiological saline, which corresponded to 10^8^ colony forming units (CFU)/mL bacterial density and a standard of 0.5 on the MacFarland scale. A suspension of 5 × 10^5^ CFU/mL was prepared with a 200-fold dilution. The germ-count of the suspensions was tested with inoculation to agar plates and counting the number of CFUs. For the determination of MIC values of florfenicol, two-fold dilution was prepared from the stock solutions on 96-well microplates with BHI. Two-fold dilution with BHI was prepared in each column to achieve working solutions with the respective final concentrations of florfenicol (0.0625–32 µg/mL). Positive control wells contained only BHI broth inoculated with the certain strain. Negative control wells contained BHI broth without inoculation. After inoculation of bacteria, microplates were placed in thermostat with a temperature of 37 °C, 0.5% CO_2_ for 24 h. The minimum inhibitory concentration was considered as the lowest drug concentration that caused complete growth inhibition. Those strains with minimum inhibitory concentration values higher than 32 µg/mL were re-tested using a broader range of florfenicol dilutions. 

### 2.6. In Vitro Killing-Time Curves of Florfenicol against SS3

SS3 was isolated for arthritis of swine, and it was an *S. suis* serotype 2 strain. SS3 was selected to be used for PD analysis, including bacterial growth and killing-time curves, in BHI liquid broth. In the bacterial growth and killing-time curve study, the preparation of SS3 suspension was similar to that described in Section 2.5. BHI test tubes were prepared at different concentrations of florfenicol, ranging from 1/2 to 2 MIC. In this part of the study, 1/2 MIC, 1 MIC and 2 MIC concentrations were equal at 0.5, 1 and 2 µg/mL. CFU counting was performed at 0, 2, 4, 6, 8, 10, 12 and 24 h. This part of the study was deemed necessary to confirm whether a verified arthritis-isolated *S. suis* behaves similarly in the in vitro killing-times test as compared to previous results [3]. 

### 2.7. Pharmacokinetic/Pharmacodynamic Integration

Lei et al. [3] established PK/PD integration modeling (incl. AUC_24h_/MIC) of florfenicol against *S. suis* for ex vivo investigations; this was followed in our study, extrapolated to synovial samples. Lei et al. [3] presented ex vivo PK/PD integration of florfenicol in swine plasma against *S. suis*. AUC_24h_/MIC ratios of 37.89, 44.02 and 46.42 were used for calculating the necessary maximum MICs respective to bacteriostatic, bactericidal and eradication effects. The bacteriostatic effect (E = 0) was achieved where the PK/PD breakpoint of AUC/MIC reached 37.89 µg/mL·h. This ratio achieved the bacteriostatic effect with florfenicol; our dosage regimen could establish an average plasma concentration of 37.89/24 h = 1.58 fold the MIC of *S. suis*. In the case of bactericidal effect (E = −3), the AUC/MIC ratio of 44.02 µg/mL·h ensured this effect with florfenicol; our dosage regimen established an average plasma concentration of 44.02/24 = 1.83 fold the MIC of *S. suis*. Furthermore, for the case of eradication, the average plasma concentration of florfenicol over 24 h in a steady-state condition should be 1.93 fold the MIC of *S. suis* [3]. After the average plasma and synovial concentrations of florfenicol were calculated in a steady-state condition, when the loading dose followed by the maintenance dose (15 mg/kg_bw_) was used to calculate the critical MICs, we could predict the bacteriostatic, bactericidal and eradication effect. In our study, we applied the recommendations of Toutain et al., where PK/PD integration is computed by the average plasma and synovial concentration of florfenicol [32]. The PK/PD index was calculated using only the reduction of 1−log (E = −1) because it was adequate to ensure efficacy as reported by Nielsen et al. [22] and Toutain et al. [33]. Lei et al. [3] did not report 1-log of reduction for the AUC/MIC breakpoint. The required average plasma concentration of florfenicol is shown Section 3.4. 

## 3. Results

### 3.1. Pharmacokinetic Parameters of Florfenicol in Plasma and Synovial Fluid after Intramuscular Administration

**Figure 1:**
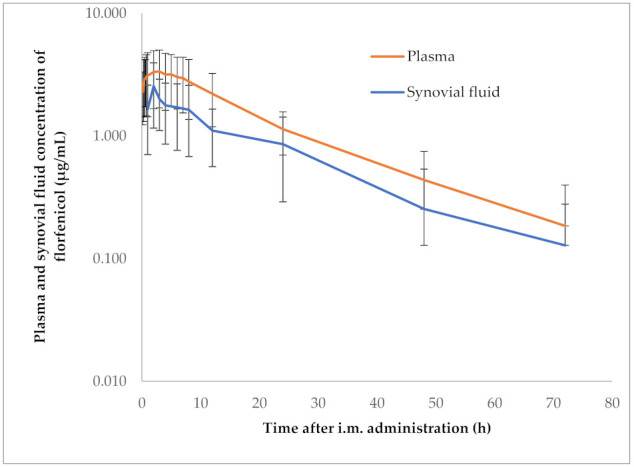
Semi-logarithmic graph illustrating the time-concentration curve of florfenicol in plasma and synovial samples of pigs after a single intramuscular administration of 15 mg/kg_bw_ (n = 6).

No abnormalities, such as irritation, sign of pain or lameness, were detected in pigs following the administration of florfenicol. The semi-logarithmic plasma and synovial fluid concentration vs. time curves of florfenicol after single i.m. administration of 15 mg/kg_bw_ are illustrated in Figure 1, and mean PK parameters are shown in Table 2. Florfenicol reached a C_max_ of 3.58 ± 1.51 µg/mL at 1.64 ± 1.74 h in plasma and reached a C_max_ of 2.73 ± 1.2 µg/mL at 3.4 ± 1.67 h in the synovial fluid of swine. The PK parameters obtained from non-compartmental analysis were AUC_0-∞_ in plasma 87.15 ± 31.48 µg/mL·h and in synovial fluid 54.8 ± 13.35 µg/mL·h, AUC_24h_ in plasma 54.66 ± 23.34 µg/mL·h and in synovial fluid 31.24 ± 6.82 µg/mL·h, Cl/F in plasma 0.19 ± 0.08 L/h/kg and in synovial fluid 0.29 ± 0.08 L/h/kg and MRT_0-∞_ in plasma 24.0 ± 13.59 h and in synovial fluid 27.39 ± 17.16 h. The V_ss_ of 4.26 ± 2.24 L/kg in plasma was calculated by 0.19 ± 0.08 multiplied by 24.00 ± 13.59 h (V_ss_ = Cl/F × MRT). Therefore, the target concentration of florfenicol of 3.63 ± 1.31 µg/mL in plasma was computed as 87.14 ± 31.5 µg/mL·h divided by 24 h (target concentration = AUC_0-∞_/24 h) in the steady-state condition. The average plasma concentration of florfenicol equaled the target concentration. Hence, a loading dose of 15.00 ± 8.49 mg/kg_bw_ could be calculated as 3.63 ± 1.31 µg/mL multiplied by 4.26 ± 2.24 L/kg (LD = C_24av_ × V_ss_). Furthermore, the synovial fluid-to-plasma ratio of florfenicol of 0.66 ± 0.13 was calculated as 54.8 ± 13.35 µg/mL·h divided by 87.14 ± 31.5 µg/mL·h (AUC_0-∞_ in synovial fluid/AUC_0-∞_ in plasma) in the steady-state condition. Consequently, the average synovial fluid concentration of florfenicol of 2.28 ± 0.56 µg/mL was computed as 3.63 ± 1.31 µg/mL multiplied by 0.66 ± 0.13 (C_av24_ in synovial fluid = C_av24_ in plasma × No. of ratio).

### 3.2. Minimum Inhibitory Concentration of Florfenicol against S. suis

**Figure 2:**
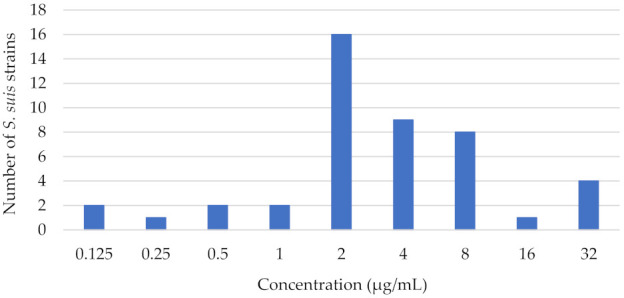
Minimum inhibitory concentration distribution of florfenicol against *S. suis* in Hungary between 2018 and 2021 (n = 45).

The minimum inhibitory concentrations of 45 isolated *S. suis* ranged from 0.125 to 32 µg/mL. The distribution of florfenicol MICs against isolates of *S. suis* is shown in Figure 2. The MIC_50_ value was 2 µg/mL; florfenicol at this concentration was able to inhibit the growth of 23 of 45 isolates. The MIC_90_ value was 16 µg/mL; florfenicol at this concentration was able to inhibit the growth of 41 of 45 isolates. 

### 3.3. In Vitro Killing-Time Curves of Florfenicol against SS3

**Figure 3:**
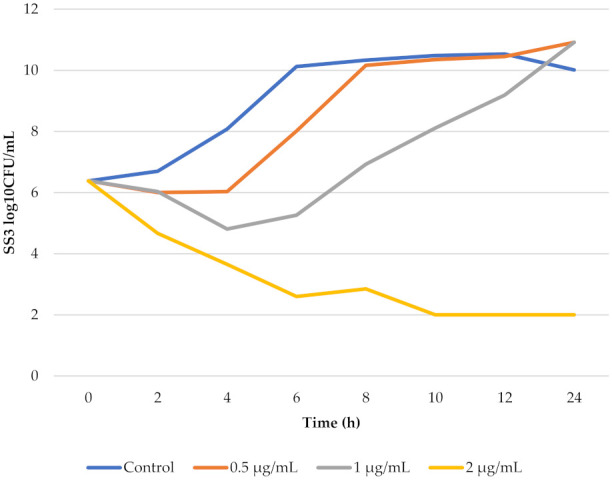
The bacterial growth-time curve and the in vitro killing-time curve of florfenicol against SS3 in BHI liquid broth. In the control, no florfenicol was added, and 0.5 µg/mL, 1 µg/mL and 2 µg/mL were equal 1/2 MIC, 1 MIC and 2 MIC values, respectively.

The MIC of florfenicol against the investigated SS3 strain was 1 µg/mL in BHI. The logarithmic phase of SS3 in BHI liquid broth occurred at an interval between 2 and 6 h. At 2 µg/mL florfenicol concentration, bacterial killing occurred. A 4-log decrease was noted after 10 h, and no recolonization was observed. The bacterial growth-time curve in BHI and the in vitro killing-time curves of florfenicol against SS3 are shown in Figure 3. 

The SS3 count reduction was 3-log when concentration of florfenicol was two-fold of MIC of florfenicol against SS3.

### 3.4. Pharmacokinetic/Pharmacodynamic Integration

Based on the AUC/MIC breakpoint specified by Lei et al. [3] and suggestions from Toutain et al. [32], the average plasma concentration required of florfenicol in fold of the MIC of *S. suis* (1.58; 1.83; 1.93) was calculated. If the bacteriostatic effect of florfenicol is to be reached in swine plasma, a 1.58-fold concentration the MIC of *S. suis* is required. In our case, this could be achieved if MIC values of florfenicol against *S. suis* were equal to or lower than 2.30 µg/mL. Hence, the average plasma concentration of florfenicol was 3.63 ± 1.31 µg/mL in the steady-state condition. The C_av24_ of florfenicol in plasma can be ensured with a dosage regimen of 30 mg/kg_bw_ (loading dose) followed by a 15 mg/kg_bw_ (maintenance dose) daily i.m. administration. For the same dosage regimen, the C_av24_ of florfenicol in synovial fluid proved to be 2.28 ± 0.56 µg/mL; therefore, the MIC values of florfenicol against *S. suis* should be equal or lower than 1.42 µg/mL. Under the same condition, if the bactericidal or eradication effect is to be ensured in plasma, MIC values should be equal or lower than 1.96 µg/mL and 1.86 µg/mL, respectively; in the same case in synovial fluid, these were 1.22 µg/mL and 1.16 µg/mL, respectively. The critical MIC calculated with the AUC_24h_/MIC values from Lei et al. [3] and our average plasma and synovial fluid concentrations of florfenicol are shown in Table 3. In this case, a reduction of 1-log was ensured between 1 µg/mL and 2 µg/mL MIC values if our dosage regimen was used.

## 4. Discussion

Florfenicol is a broad-spectrum antibacterial agent, mainly used in cattle and pigs via i.m. injection and in poultry and fish via drinking water administration [1,34]. This study aimed to evaluate the use of florfenicol in pigs against *S. suis* septic arthritis based on PK/PD integration. Florfenicol was administered intramuscularly to six pigs at a single dosage of 15 mg/kg_bw_, resulting in the pharmacokinetic parameters in synovial fluid as shown in Table 2. Our study was the first to describe the pharmacokinetic parameters of florfenicol in synovial fluid in swine. Our results were obtained in healthy pigs, but Errecalde et al. [35] observed that the amoxicillin concentration in synovial fluid in arthritic horses was higher and more persistent than in healthy ones. Therefore, florfenicol is more likely to achieve higher concentrations in synovial fluid in arthritic swine. The pharmacokinetic properties of florfenicol in synovial fluid after intravenous regional perfusion was studied previously in healthy cows, where the following values were obtained: C_max_ was 39.2 µg/mL and T_max_ was 48 min after administration [4]. In another study, lower synovial fluid concentrations in cattle were reported to be counteracted by a shorter half-life of florfenicol in synovial fluid of 65 h as compared to plasma, where the elimination of the drug from the synovial fluid was slower than 38 h, and the concentration remained above 0.5 µg/mL for 72 h, which is a potential benefit for the treatment of joint pathogen bacterial strains in cattle [7]. In the present study, C_max_ and T_max_ values of florfenicol in blood plasma were found to be almost similar to the results (3.04 μg/mL, 1.94 h) documented by Dorey et al. [36] in pigs after administration via the intramuscular route (15 mg/kg_bw_) but lower than the levels obtained by Voorspoels et al. [6] (7.3 μg/mL, 2.3 h) in pigs after administration via the intramuscular route (20 mg/kg_bw_). Therefore, if florfenicol is administered in a higher dosage, it will also be present at a higher concentration in synovial fluid in swine.

*S. suis* is one of the most important swine pathogens worldwide, causing severe economic losses to the pig industry and responsible for losses of more than $300 million per year in the United States alone [16]. Due to the increasing prevalence of antibiotic resistance, antibacterial therapy against infections should be based on the results of antibiotic susceptibility testing of the pathogen, preferably including the determination of MIC values. In this study, MIC values of florfenicol were investigated in 45 *S. suis* strains collected in Hungary. The MIC_50_ and MIC_90_ values were found to be 2 µg/mL and 16 µg/mL, respectively, and the distribution of MIC values was similar to previous results. In the Netherlands, between 2013 and 2015, 1163 *S. suis* strains were investigated, and both the MIC_50_ and MIC_90_ values of florfenicol were ≤2 µg/mL against the isolates [37]; the same results were obtained in an EU monitoring project, where MIC, MIC_50_ and MIC_90_ values were determined in 151 *S. suis* isolates between 2009 and 2012 [38]. Furthermore, the same MIC values were described in England between 2009 and 2014 [39]. Similar results were obtained in the United States and Canada between 2011 and 2015, where 1201 samples were analyzed [18]. Our results showed similarity to previous results in the distribution of MICs and MIC_50_, but MIC_90_ values were significantly higher in Hungary. The presumed reason for the unfavorable MIC_90_ values may be the high antibiotic use in Hungary, as reported by the European Surveillance of Veterinary Antimicrobial Consumption in 2018 [40].

The matrix effect plays a crucial role in the determination of the MIC and in vitro killing-time curve. More preciously, based on a review by Toutain et al. [33], the medium can have a positive or a negative effect on the MIC value. In our case, based on the study of Lei et al. [3], the MIC value of florfenicol against *S. suis* is higher in broth medium than in porcine serum. It can therefore be assumed that, within species, in the synovial fluid of pigs, the MIC value might be lower. Therefore, in our study the broth medium was a so-called worst-case scenario from a clinical aspect. The in vitro killing-time curves of florfenicol against SS3 obtained in our study (Figure 3) were similar to the results of Lei et al. [3]. Consequently, their ex vivo results were used as the basis of our PK/PD integration, from which the following results were obtained. Presumably, florfenicol treatment via intramuscular administration with a loading dose of 30 mg/kg_bw_ first day of treatment, followed by maintenance dose of 15 mg/kg_bw_ once daily, should be bacteriostatic against *S. suis* in the synovial fluid of pigs if the MIC value of the strain is equal to or lower than 1.42 µg/mL. Toutain et al. [33] advised that E = −1, used in human medicine for non-immunocompromised patients, is sufficient in veterinary PK/PD calculations as well [22]. In this case, the treatment of florfenicol against *S. suis* should be satisfactory if the isolate has an MIC of about 1–2 µg/mL. This susceptibility of *S. suis* is similar to the recommendation of CLSI for respiratory tract infections of *S. suis*. On the other hand, we can assume that in acute arthritis caused by *S. suis*, the concentration of florfenicol in synovial fluid is higher. In these cases, *S. suis* with higher MIC values may also be susceptible, but this requires further investigations. In addition, the important justification for our study is that currently there is no formulation containing florfenicol authorized for arthritis in swine caused by *S. suis* at a dosage regimen similar to our findings. As a consequence of the latter, the application of a new dosage regimen has to take into account the country and region regulations, e.g., the withdrawal period or veterinarian responsibility. These reasons led to the hypothesis that florfenicol might be a potential candidate for treatment of arthritis in swine caused by *S. suis*.

In conclusion, based on our study, florfenicol treatment (after i.m. administration of a loading dose of 30 mg/kg_bw_ followed by a maintenance dose of 15 mg/kg_bw_) might be an available treatment option for arthritis in swine caused by *S. suis*, but it is highly dependent on the MIC of the pathogen. Consequently, our dosage regimen can only be proposed based on an antimicrobial-susceptibility test, which proves that the MIC of florfenicol against *S. suis* is equal to or lower than 2 µg/mL. The results in our study are based on averages, and the Monte Carlo simulation was not calculated. 

This study is the first presentation of the pharmacokinetic behavior of florfenicol in swine synovia and a recommendation of the extrapolated critical MICs of *S. suis* for therapeutic success in the treatment of *S. suis* arthritis in swine. Target tissue pharmacokinetics and PK/PD analysis is fundamental to the evidence-based treatment of bacterial diseases in veterinary medicine, and as such, facilitates prudent antimicrobial use and the global reduction of AMR in the animal industry.

## Figures and Tables

**Table 1:** Formula for calculation of loading dose, daily dose and target.

**Plasma**	In steady-state condition in plasma **AUC_24h_ = AUC_0-∞_** **LD = V_ss_/F × target concentration** **V_ss_ = Cl/F × MRT** **target concentration = C_24av_** in steady-state condition in plasma **C_24av_ = AUC_0-∞_/24 h**	**LD = C_24av_ × V_ss_**
**Synovial fluid**	**Ratio = AUC_0-∞_ of plasma / AUC_0-∞_ of synovial fluid**	
	**C_24av_ of synovial fluid = No. of ratio × C_24av_ of plasma**	

AUC_24h_: area under the curve for 24 h; AUC_0-∞_: area under the concentration-time curve from zero time to infinity; LD: loading dose; V_ss_/F: steady-state volume of distribution scaled by bioavailability; Cl/F: drug clearance scaled by bioavailability; MRT: mean residence time; C_av24_: average plasma concentration.

**Table 2:** Plasma and synovial fluid PK parameters (mean ± SD) of florfenicol in pigs following intramuscular administration of 15 mg/kgbw (n = 6).

Parameters	Unit	Plasma	Synovial Fluid
C_max_	µg/mL	3.58 ± 1.51	2.73 ± 1.2
T_max_	h	1.64 ± 1.74	3.4 ± 1.67
T_1/2_	h	17.24 ± 9.35	21.01 ± 13.19
AUC_24h_	μg/mL·h	54.66 ± 23.34	31.24 ± 6.82
AUC_0-∞_	μg/mL·h	87.14 ± 31.50	54.80 ± 13.35
Cl/F	L/h/kg	0.19 ± 0.08	0.29 ± 0.08
MRT_0-∞_	h	24.0 ± 13.59	27.39 ± 17.16

C_max_: maximum plasma concentration; T_max_: time to peak plasma concentration; T_1/2_: terminal elimination half-life; AUC_24h_: area under the curve for 24 h; AUC_0-∞_: area under concentration-time curve from zero time to infinity; Cl/F: drug clearance scaled by bioavailability; MRT_0-∞_: mean residence time.

**Table 3:** Mean indicative MIC breakpoints of florfenicol against *S. suis* for bacteriostatic, bactericidal and eradication effects. The required average plasma concentration should be a multiple of the florfenicol MIC against *S. suis*. Critical MICs of *S. suis* in synovial fluid and plasma were calculated based on the breakpoints of Lei et al. [3]. Our AUC_0-∞_ in plasma and synovial fluid (87.14 ± 31.50 µg/mL, 54.80 ± 13.35 µg/mL) was divided by breakpoints of Lei et al. [3] (37.89, 44.02, 46.42).

Effect	^1^ AUC_24h_/MIC Breakpoints	Average Plasma Concentration Required (in Fold of the MIC)	MIC of *S. suis* in Synovial Fluid (µg/mL)	MIC of *S. suis* in Plasma (µg/mL)
**Bacteriostatic**	**37.89**	**1.58**	**≤1.42**	**≤2.30**
**Bactericidal**	44.02	1.83	≤1.22	≤1.96
**Eradication**	46.42	1.93	≤1.16	≤1.86

**^1^** Lei et al. [3].
